# Baltimore Medical Association

**Published:** 1883-10-20

**Authors:** 


### BALTIMORE MEDICAL ASSOCIATION.

### A STATED MEETING HELD THE 26TH OF MARCH, 1883.

The Association was called to order by the President, Dr. J. S. Conrad, at 8:30 p. m., in the presence of twenty-three members.

The committee of honor reported favorably on the names of Drs. C. D. Smith and John N. Mackenzie, who were then elected to membership.

Drs. Z. K. Wiley and S. J. Fort were proposed for membership.

Dr. Cordell reported the following case of "Adherent Placenta following miscarriage, accompanied by alarming hemorrhage, necessitating its forcible removal."

A young unmarried girl, æt. 19, who had missed her monthly sickness, according to her account, for two months, began to have a slight discharge of blood February 6th. This had ceased by evening, to return more freely two days later. She passed a large quantity of blood, both fluid and in clots, until the night of the 9th, when severe intermittent pains in the back and abdomen set in. Being called on the following morning Dr. C. found the os firmly contracted. Not being able to find out whether any portion of the ovum had been passed or not, opium was given freely with the object of arresting, if possible, the pains, and preventing miscarriage. The pains were relieved but the oozing of blood continued copiously, so that at night the patient was much exhausted. The os was now softer and patulous and clots of blood protruded from it. Deeming the miscarriage now inevitable, and fearing the effects of further hemorrhage, he proposed introducing a tampon, but the patient obstinately refused to allow it to be done. Accordingly she was left for the night as she was. On the following morning she was very weak and anæmic, suffered with giddiness on motion. with nausea and vomiting, fluttering pulse becoming very rapid on the least exertion.

Her dangerous condition was now fully explained to her, with the statement that unless she consented to being treated as he thought best he would resign the case. Upon these representations she consented, and he proceeded to empty the uterus, after having given two drachms of Squibb's fluid extract of ergot at intervals of a half hour.

During the pains excited by this agent, he introduced two fingers of the right hand and found the os soft and patulous, and the supposed blood clots protruding from it. He had no difficulty in reaching the cavity of the uterus, and by pressing down the abdominal walls (the patient being under the influence of chloroform) detaching and withdrawing the placenta, which he found quite firmly attached to the fundus. Having thus emptied the uterus he passed a sponge tampon dipped in vinegar up to the os. The after-birth, which seemed, by its size, to indicate a more advanced period of pregnancy than corresponded with the patient's statement, was found in fragments in the bed among the blood clots. The sponge was removed in a few hours, and it was found that the os was contracting and there had been no hemorrhage. The patient is now doing well. (Under the use of the tinct. ferri chloridi, whisky, milk and beef tea, she made a rapid recovery, and was up on the ninth day. *Rep.*)

Dr. Erich spoke favorably of the suggestion made by Dr. Williams, viz: to give small doses of ergot frequently for the purpose of arresting hemorrhage in threatened miscarriages. He had tried it in the case of a lady, two months pregnant, who had had a large hemorrhage, giving ten drops every hour until the hemorrhage ceased, then at longer intervals. She continued using the remedy thus—having recurrence of the hemorrhage at intervals—until the fifth month. Then, in consequence of the loss of her father, she miscarried twin children. She had, meanwhile, taken a large quantity of the remedy. Although he failed to accomplish his object of carrying her to term, he felt confident that her pregnancy had been protracted by the treatment. The case shows that ergot will arrest hemorrhage occurring under such circumstances, and that it is safe. He has used it repeatedly and always with satisfaction.

Dr. Ellis thought that in recurring hemorrhage without pain it would be safe and efficient, but where pains are marked it would be a very dangerous agent.

Dr. Erich said hemorrhage causes abortion by filling the cavity of the uterus and exciting contractions. There is then alternate relaxation and contraction; the small doses of ergot render the contraction continuous.

Dr. Browne said a woman having a fall and then threatened abortion demands opium, not ergot. The rule is with much pain and cervix contracted, give anodynes, if there be flooding, patulous cervix and considerable hemorrhage, give ergot. In some cases we unite the latter with opium or viburnum prunifolium. In many cases of abortion ergot is not applicable.

The President observed that ergot has two distinct effects, one its specific action on the uterus; the other, its vaso-motor or hemostatic action. The small doses spoken of, therefore, may produce the latter, and not the specific.

---

"Phthisis Arrested by an Attack of Typhoid Fever."—Dr. Gilman reported the case of a young lady predisposed to phthisis, her father and mother both having died of it. She went to Europe after the signs of phthisis had developed in her, had typhoid fever in Rome, and came back apparently well.

Dr. Gilman then referred to a letter received from Dr. Buckler, of Paris, in which the writer takes the view that typhoid fever is prophylactic against phthisis.

By vote Dr. Gilman was requested to present Dr. Buckler's letter to the Society at the next meeting.

---

"What Shall we do with Chloroform"?—Dr. Jos. T. Smith opened the discussion of this subject with a paper, which has been published in the Southern Clinic. The conclusion of the paper was in favor of chloroform. In children and obstetrics it is not in question, but only in surgical practice. It is far superior to ether in its action and effects. Ether kills in but one way, chloroform in two.

Dr. Rohe said it was a mistake to suppose that chloroform is entirely safe in children. Ten per cent. of all the cases of death under the anæsthetic are observed in children under twelve. He believed that the proportion would even reach one-half if the ratio of children to adults operated upon were taken in consideration. The safety of chloroform in labor depends upon two causes: 1st. It is rarely given to complete anæsthesia. 2nd. There are good grounds for believing that there is hypertrophy of the heart at this time, as pointed out by Dr. Fancourt Barnes.

Dr. Erich said one reason of the opposition to ether is that very few know how to use it. He had used it for twenty years, and uses it alone. Has not seen any ill effects from it. At first he employed the cone formed by a towel, when a half hour was required to produce anæsthesia, the room was filled, and the operator became nearly as drunk as the patient. He used chloroform in labor, and regards it then as safe, the excitation and pain producing a tolerance of it.

By excluding air we get the patient under the influence of ether in three minutes—on the average quicker than chloroform. In a case of vesico-vaginal fistula only a little over two ounces were used. Dr. Erich regularly employs Rohe & Leonard's India-rubber bag, with mouth-piece. The patient must be prepared for a feeling of suffocation at first, and his hands must be held for a few breaths. If he understands fully what is being done he will co-operate. After he gets under the influence of the agent we may give air as freely as we please. Here we may have vomiting after the operation, but not during it. In chloroform the reverse is the case. The opposition would cease if the proper method of using ether were known and practiced.

Dr. Waters had been present at about 1,000 administrations of chloroform. The only case of a threatening character from the use of anæsthetics was when ether was used. In that case the patient had been sinking.

Dr. Rohe quoted Lyman's statistics, according to which 104 died before the full effect of the chloroform was secured, 105 after. It is a mistake to suppose that death always occurs at the beginning of the anæsthesia.

Dr. J. T. Smith replied that authorities, as Stille, state that chloroform is safe in childhood. The struggling by ether is not to be prevented. In chloroformization, vomiting may be prevented by not allowing food for some hours before anæsthesia.

The President said on the battle-field chloroform was given with the utmost feeedom, and there was never any bad result. It was only when he got into civil practice that he began to hear of bad results. But in twenty years' practice (in which he had given it once a week on an average) he had experienced bad results in but two cases, viz: In a boy who had strangulated hernia, for which taxis was being employed, and who became rigid and collapsed, but was restored by prompt treatment; in a case of fistula where the respiration and heart-beat ceased but were restored by treatment.

One of the greatest recommendations of chloroform is the small bulk required, one ounce usually sufficing.—*Maryland Medical Journal.*

